# Medical Society of the County of Albany

**Published:** 1871-04

**Authors:** James S. Bailey


BUFFALO

### Medical and Surgical Journal.

VOL. X. APRIL, 1871. No. 9.

### Original Communications.

ART. I.—*Medical Society of the County of Albany. Semi-monthly Meeting, March 14th*, 1871.

REPORTED BY JAMES S. BAILEY, M. D.

Dr. ANDREW WILSON, Vice-President, in the Chair.

*The Physiological Action and Therapeutical Effects of Codeine.*—Dr. J. W. MOORE remarked, codeia exists in opium, combined with meconic acid, and is extracted with that alkali in the preparation of the muriate of morphia. It is obtained by treating with ammonia a solution of codeia and murriate of morphia, the latter being precipitated while the codeia remains in solution. There is some danger in its preparation of its containing more or less of the stronger alkaloid, and frequently unmistakable evidences of morphia are noticed after its administration. Its presence may be detected by the tests of nitric acid or sesqui chlor ferri: the former rendering the blood red color and the latter the blue solution.

Dr. Moore was first led to the use of codeia, about three years ago, in a case of acute dysentery in a female, aged 30, of nervous temperament. It was a case of consultation. All the other remedies had been tried that could be thought of with no apparent relief. There were present bearing down pains, frequent blood and mucous stools, with violent tenesmus, and also obstinate vomiting. She was unable to keep anything upon her stomach, no matter how bland. She had been confined to her bed for about ten days, and for the last three, had been unable to take food or nourishment of any kind. It was impossible to take a little water without its immediate rejection.

She could not tolerate opium, or any of its preparation, by the mouth, to relieve the excessive pain. Opium, by injection, was tried, but it only increased the nausea and produced troublesome headache. At last two grains of oxalate of cerium was dropped dry upon the tongue, the administration of which was soon followed by a cessation of retching, so that in the course of an hour she was enabled to take and retain a grain of codeia in solution, which was soon followed by a quiet, sweet sleep, lasting some hours. Dr. Moore anticipated nausea upon awaking, but it did not return again. The headache was entirely relieved; her bowels before had been exquisitely tender and tense, were now quite flaccid, and could bear gentle pressure. The sulphate of magnesia was administered, which produced a fœcal stool. Codeine and cerium were continued for several days, and the quiet repose that had followed its first administration was always produced. There was no constipation, increased heat or frequency of the pulse following. The patient was soon able to retain food, and soon recovered.

The doctor had treated fifteen or twenty cases by the use of this remedy, and had noticed turbity and redness of the urine; but, having never administered the drug in any cases excepting those that were suffering from disease, he is unable to state whether the deposit was the result of the action of the medicine or of the disease.

Persons are relieved of dysentery by this remedy, when the stomach can contain it. We occasionally have a disease of the bowels called nervous dysentery, which originates from some particular derangements of the great sympathetic, and not from any climactic or zymotic cause, which seems to be unmanagable by the ordinary remedies, in consequence of extreme nervous excitability of the stomach. From experience Dr. Moore thinks codeine particularly applicable in such cases, and when judiciously administered will effect a speedy cure. He had never given more than two grains at a dose; and, when the drug was pure, had never observed the unpleasant after effects indicating the presence of morphia in sufficient doses to procure sleep.

*Bromide of Potash as an Anthelmintic.*—Dr. J. N. NORTHROP

gave the history of the following interesting case. In 1870 Mr. Smith called upon the Doctor to consult him for the relief of tape worm. He had upon several occasions passed segments of the same. The usual symptoms were manifest: distended abdomen, peaked countenance, &c., wnich had been of two or three years duration. He had used the ordinary remedies, spits. turpentine in large doses, pumpkin seed, &c., without relief. Bromide of potash in twenty grain doses, every four hours, was prescribed, to be used until the sedative effects was experienced. He continued the remedy two and a half or three days, then took half an ounce of spirits turpentine, and in a short time two ounces of castor oil. The Doctor heard no more from his patient for several weeks, until he was congratulated by a neighbor of his patient, who assured him he had made a remarkable cure. That, from the first medicine, he had passed in all 200 feet; and after a few weeks, had repeated the medicine, and passed 50 feet more, at which time the head was voided, making in all 250 feet of tape worm. Mr. S., since that time, had rapidly improved in health, and had not since been troubled with the parasites.

Dr. Northrop was since attending a child between two and three years old. He gave bromide of potash in syrup, in sedative doses, when twelve feet of tape worm were passed. The child improved in health and has remained healthy since.

Dr. Northrop had recently prescribed the remedy for tape worm, in the case of an adult, but had not heard of the result. He hoped the members of the society would give the bromide of potash a trial if such cases were presented for treatment, and report the result to the society.

*Cases of Apoplexy occurring after Labor.*—Dr. JAMES S. BAILEY reported the following cases:

CASE I.—In 1867 I was called, at 10 o'clock, P. M., to attend Mrs. D., a healthy looking Irish woman, in her ninth accouchment. Her pains were occurring strongly, at intervals of five minutes; by 3, A. M., she was delivered of a large sized male child. He remained to see that the umbilicus was properly dressed, which consumed about half an hour; during this time she was cheerful and sociable, remarked upon her unusually hard labor, and attributed it mostly to her being in better flesh than usual. In the course of an hour from this time she fell asleep, and rested quietly until about 9 A. M., when she was noticed to breathe stertorously. An effort was made to awaken her; as it was not successful, a messenger was sent for Dr. B. By 11 o'clock he saw her; she was in a deep coma, with the pupils of her eyes contracted, countenance livid and somewhat turged. In half an hour she expired. No post mortem was allowed.

This woman was accustomed to work out by the day to wash and clean house. She had been thus engaged upon the day of her confinement. There is no doubt but during the severe uterine contractions one of the blood vessels of the brain was ruptured, which caused a gradual extravasation of blood, which caused this deep sleep—this apoplexy.

Case II.—Mrs. A, aged 35, an apparently healthy woman, weighing about 140 lbs. She had three living children, and three premature births. He was called in a threatened case of miscarriage, October 22d. The hemorrhage was considerable, and the os but slightly dilated, without having experienced labor pains. The annoying symptoms being excessive nausea and pain in the gastric region.

The next day the gastric distress was not relieved, the hemorrhage was considerable, with an increase in labor pains and a dilatation of the os uteri. At 3½ P. M. he was again summoned in haste, and ascertained at 3 o'clock she had been delivered of a small sized male infant, appearing as if she was four months advanced, though she had claimed to have passed her seventh month of conception.

About fifteen minutes after delivery she attempted to raise her head, complained that she could not see, and heavily sank back upon the pillow, and was immediately insensible. This was her condition when Dr. B. arrived. Dr. Wm. H. Bailey was passing and was invited to see the case with him. Her pulse was forty-five per minute, and not strong. Her face was flushed and mottled, her eyes closed, and the balls turning upwards, the pupils much contracted, breathing was slow but not stertorous. There was frequent retching, and a dark foamy mucus was vomited. He considered it a case of apoplexy. In about three hours they again saw her. The circulation had improved, and the skin assumed a normal appearance; deglutition was exceedingly difficult. Upon the evening of the second day the pupils were much dilated and paralysis developed upon the right side, though she did once or twice partially raise her right arm. Her bowels responded readily to a moderate dose of castor oil. The urine was involuntarily discharged.

Her condition apparently improved, her intellect brightened, she followed him with her eyes when passing around her, would raise her right leg when requested, and nod her head in answer to questions but could not articulate.

Upon the morning of the fourth day there was a perceptible change for the worse: there was more insensibility, and the breathing was shorter and more hurried. Upon the evening of the fifth day she expired without a groan.

*Autopsy*—twenty-one hours after death.

*Body*—well nourished, rigor mortis present, and a sanguinous fluid oozing from the mouth ; considerable sugillations over posterior part of trunk.

*Head*—skull cap normal. The dura mater being removed, the vessels of the arachnoid were much injected and engorged with blood. A small quantity of serum was contained in the sub-arachnoidian cavity. Upon removing the cerebrum with the cerebellum and medulla oblongata, a considerable quantity of blood was found effused over the anterior surface of the medulla oblongata, and the fourth ventricle was filled with a clot of blood which extended into the third ventricle, and was continuous with a very large cloth which occupied both lateral ventricles, more especially that on the left side. The superior portion of the left corpus striatum was softened and lacerated by the effusion of blood. The remainder of the brain tissue appeared normal.

*Thorax.*—The right lung was adherent to the ribs by an old and extensive adhesion. The posterior and inferior portions of both lungs were engorged with blood ; indurated and easily torn portions cut from these parts did not float in water. Heart, pericardium and valves presented nothing abnormal.

*Abdomen.*—No peritoneal effusion ; the viceral and uterine peretoneum was red, and the vessels injected. The uterus measured seven inches in length, four and one-half inches in breadth, and two inches in thickness, and contained a dark, friable clot of blood.

The left kidney was pale and mottled upon its surface; upon the capsule free and extravasated blood was found on its surface at points corresponding to the mottled appearance. The right kidney was of normal size, and did not present the mottled look. Both kidneys were in a fatty degeneration. The liver was normal in size, and of a dark olive color and soft.

*Cases of Hysteria terminating in Apoplexy.*—Dr. JAMES S. BAILEY reported the following:

CASE III.—See "Buffalo Medical and Surgical Journal," Vol. IX., No. 10, page 371.

CASE IV.—Mrs. McN., aged 35, a spare built woman, the mother of eight children, the oldest nineteen, youngest six years of age—since the birth of which she has had two miscarriages. Her catamenia had appeared regularly until her last period, since which time six weeks had elapsed, when she again became "unwell," and continued to flow her usual length of time, seven days. After she ceased to flow she was seized with hysteria, with a manifestation of its various symptoms. It was in one of those paroxysms the Doctor was called to see her. He found her laying upon a lounge screaming violently, perspiring moderately, with cool extremities, pulse and temperature normal. Her eyes presented a peculiar bleached and faded color; the pupils were contracted; there was intolerance to light, and much palor of countenance. All efforts to restrain her cries were unavailing; she complained only of a strange sensation in the top of the head. She was apparently relieved and quieted by the use of hydrat chloral, in 10 grain doses, together with mustard pedaluvia. During half the night she rested quietly, and in the morning was much relieved, but complained of numbness of her extremities.

Having witnessed a similar case (No. 3) before, which terminated in apoplexy, he feared this might also so terminate, and expressed this belief to the family, which was unfortunately realized within a space of three hours. Soon after she was seized with a convulsion, and remained insensible with stertor until she died.

No *post-mortem* was allowed.

*Cases of Hysteria resembling Apoplexy.*—Dr. JAMES S. BAILEY also reported the following:

CASE V.—Several years ago he was called in haste to see a young lady of fine personal appearance, who was found in her room insensible. At first the impression was, that she had swallowed poison. She lay passively upon the bed; there was no ridgity nor spasms of her limbs; pulse and temperature normal. Her eyes were closed, and when opened they presented a peculiar expression of recognition. By looking into the eye, there was no disposition to wink, nor for the eye to fill with tears. She remained in this condition three days and nights. He called several physicians to see the case with him, but council could throw but little light upon the case until an accurate history of her surroundings could be obtained. She was apparently supporting herself by sewing. Late at night an elderly man was observed occasionally to call upon her. Upon one of these visits a misunderstanding occurred, and this condition followed.

Pinching, or the prick of a pin did not cause any manifestation of pain; respiration was normal. At the expiration of the time mentioned, she gradually became conscious, and was apparently as well as usual, except debilitated from long fasting. In this case of hysteria the history of circumstances revealed more than a physical examination.

CASE VI.—About six months from this time he was called to see a young married lady, from a neighboring city, who was visiting Albany for the purpose of obtaining a divorce from her husband.

During the progress of the case some unforseen circumstance arose prejudicial to her interests, and while conversing upon the subject she fell suddenly upon the lounge, and the whole household was thrown into consternation. The circumstances, and her physical condition, so clearly approximating the case just related, he determined it, too, was a case of hysteria. Council was called, and finally her family physician. This manifestation of anxiety upon the part of friends did not serve to improve her condition, but upon the contrary to make her worse.

There was a difference of opinion among the consulting physicians. Dr. B. had already given his opinion. The consulting physicians thought the malady more grave—apoplexy. He accordingly was dismissed, and had the pleasure (?) of seeing the gentleman whom he was instrumental in having called as council supercede him, and continue to take charge of the case. Shortly before he was discharged, there was apparently paralysis developed upon one side. This case continued under treatment some time in this city, and finally returned home, still suffering from the loss of use of one side.

A few days ago a physician from her neighborhood, who was thoroughly acquainted with the circumstances, informed Dr. B. she had frequent repetitions of these attacks of apoplexy (?), but was apparently in the enjoyment of fine health.

Dr. C. A. ROBERTSON reported a very interesting case of *Preverted sense of distance from violence to the Eye.*

